# Montreal Cognitive Assessment Hearing Impairment (MoCA-H): cross-cultural adaptation to Brazilian Portuguese

**DOI:** 10.1590/2317-1782/e20240125en

**Published:** 2025-01-20

**Authors:** Rochele Martins Machado, Karina Carlesso Pagliarin, Fernanda Soares Aurélio Patatt

**Affiliations:** 1 Departamento de Fonoaudiologia, Universidade Federal de Santa Maria – UFSM - Santa Maria (RS), Brasil.

**Keywords:** Hearing Loss, Cognition, Psychometrics, Validation Study, Adaptation

## Abstract

**Purpose:**

This study aimed to adapt the Montreal Cognitive Assessment Hearing Impaired (MoCA-H) into Brazilian Portuguese (BP).

**Methods:**

This was a descriptive, cross-sectional, quantitative, and qualitative study involving participants selected by convenience. The instrument was adapted from its original version, in a six-stage process consisting of the following: Stage 1 - Translation and back translation of the MoCA-H; Stage 2 - Stimulus analysis and selection; Stage 3 - Semantic analysis of stimuli; Stage 4 - Analysis by non-expert judges, part 1; Stage 5 - Analysis by non-expert judges, part 2; Stage 6 - Pilot study. The following statistical methods were used in this study: parametric T-test, Gwet’s first-order Agreement Coefficient (AC1), and the Content Validity Ratio (CVR).

**Results:**

Cultural and linguistic adaptations were made to the instrument as well as changes to administration procedures to improve respondent comprehension. Participants with and without hearing loss had some comprehension difficulties in the visualspatial/executive domain task. This was observed not only in Stage 6 but also from the beginning of the adaptation process.

**Conclusion:**

The adaptation process yielded an instrument with satisfactory content validity.

## INTRODUCTION

Population aging is having a major impact on demographic trends. The demographic transition associated with this phenomenon has far-reaching repercussions, especially regarding age-related illnesses, resulting in major transformations in individuals and society as a whole. As a result, public policy must be adjusted to ensure that the needs of individuals aged 60 years or older are addressed^(1)^.

The aging process leads to several biological changes, with hearing loss as one of the most prevalent. Although this situation has been changing, hearing loss is still undertreated and underdiagnosed^(2)^, which is problematic given the association between untreated hearing loss and cognitive decline^(3-6)^.

Several studies have found that the presence of auditory alterations in older adults is associated with a greater risk of cognitive decline, especially in functions such as abstract reasoning and orientation^(4,5,7,8)^. Additionally, the limitations imposed by social isolation, reduced communication, and impaired autonomy, all of which may result from hearing loss, can accelerate cognitive decline^(9-11)^.

The aforementioned findings underscore the importance of assessing the impact of hearing loss on cognitive processes in individuals with hearing loss. This would also help older adults understand the importance of treatment adherence. Therefore, in these cases, standardized cognitive tests are crucial for diagnosis and effective treatment, which yields objective results and contribute to the quality of life of patients and their families^(12)^. However, inadequate protocols for the population with hearing loss can result in incorrect diagnoses, compromising the assessment's conclusion due to the existing sensory impairment^(13).^

Yet the instruments used to assess cognition in Brazil, such as the Mini-Mental State Examination (MMSE)^(14)^, Addenbrooke’s Cognitive Examination-Revised (ACE-R)^(15)^ and the Montreal Cognitive Assessment (MoCA)^(16)^, were all standardized in hearing populations. The MoCA has been widely used in the international literature to investigate cognitive impairments associated with Alzheimer’s disease^(17-19)^, Parkinson’s disease^(20-23)^, Huntington’s disease^(24-26)^, multiple sclerosis^(26-28)^, head trauma^(29-31)^, depression^(32-34)^, tumors^(35,36)^, cardiac insufficiency^(37-39)^, and COVID-19^(40-42)^, among other illnesses.

Recently, Dawes et al.^(43)^ published a version of the MoCA^(16)^ for individuals with hearing loss named the Montreal Cognitive Assessment - Hearing Impaired (MoCA-H), but the instrument was not available in Brazilian Portuguese (BP).

The MoCA-H is available in English, Dutch, German, and Italian (MoCA Cognition, n.d.) and is indicated for use in the cognitive screening of individuals 60 years or older diagnosed with hearing loss. The instrument assesses eight cognitive domains: executive functions, naming, attention, memory, abstract reasoning and orientation, late recall, visuospatial skills, and language^(43)^.

Psychometric studies are undoubtedly valuable for determining the reliability and quality of assessment instruments^(44)^. Furthermore, the authors’ familiarity with psychometric principles, combined with their knowledge of the conceptual model, assessment processes, and measurement properties of an instrument is crucial to ensure adequate and accurate results^(45)^.

Given the importance of cognitive assessment for individuals with hearing loss and the absence of an assessment protocol that evaluates the cognitive skills of BP speakers with hearing impairments, the present study was conducted to adapt the MoCA-H for the Brazilian population and analyze the performance of participants on this measure.

## METHOD

The present study was approved by the University Research Ethics Committee under number 5.162.650. The authors of the original instruments were contacted for permission to adapt the MoCA-H before the research began. As per National Health Service resolution 466/12, all participants signed an informed consent form upon entering the study.

### Participants and procedures

The study was conducted in six stages followed by an analysis of participant performance. Each stage of the study involved a different population. Participants in the adaptation process included an English teacher, a psychologist, three speech pathologists, 18 non-expert judges, and 30 neurologically healthy older adults (with and without hearing loss). Chart 1 shows the stages involved in the study with their respective samples and inclusion criteria for participation.

**Chart 1:** Stages of the study and participants inclusion criteria

Stages	Population (n)	Criteria
Stage 1:	1 Psychologist	Fluent in English and Brazilian Portuguese
Translation and back translation of the MoCA-HI	1 Speech pathologist	Translation – neuropsychology experts
	1 English teacher	Back translation – unfamiliar with the area of study
Stage 2:	2 doctorate-level speech pathologists	Responsible for the adaptation of the instrument
Stimulus analysis and selection	1 Speech pathologist studying toward a Master’s degree	
	1 Portuguese language teacher	
Stage 3:	12 non-expert judges	Absence of self-reported auditory and cognitive complaints
Semantic analysis of stimuli	(6 women, 6 men)	
		At least four years of education
Stage 4:	5 non-expert judges	Absence of self-reported auditory and cognitive complaints
Assessment by non-expert judges, part 1	(3 women, 2 men)	
		60 years and older
		At least four years of education
Stage 5:	1 non-expert judge	Absence of self-reported auditory and cognitive complaints
Assessment by non-expert judges, part 2	(1 man)	
		60 years and older
		At least four years of education
		Portuguese speaker
Stage 6:	15 PNHL	Normal hearing or (moderate to severe) hearing loss in both ears
Pilot study	(10 women, 5 men)	
	15 PWHL	Users of HADs: at least six months of hearing aid use for at least six hours a day, as confirmed by data logging
	(7 women, 8 men)	
		Normal cognition
		60 years and older
		At least four years of education
		Brazilian Portuguese speakers

Caption: HAD = Hearing Assistive Device; PWHL = Participants with hearing loss; PNHL = Participants with no hearing loss

The following sections describe the stages involved in the adaptation and validation of the MoCA-H to BP.

### Stage 1 - Translation and back translation of the MoCA-H

The translation and adaptation of the MoCA for subjects with hearing loss (MoCA-H) were conducted using the version of the adapted by Dawes et al.^(43)^

In this stage of the study, two independent translations of the instruction manual, test form, and instruction cards were obtained. The two translators involved in this process were fluent in English and experts in neuropsychology (one was a speech pathologist and the other a psychologist). The translations were consolidated into a single document which was back-translated by an English teacher with no knowledge of the field of study. The resulting version of the instrument was then sent to and discussed with the authors of the original MoCA-H.

### Stage 2 - Stimulus analysis and selection

Participants in this stage consisted of three speech pathologists - two with doctorates and one studying toward a Master’s degree - and one Portuguese language teacher. During the translation process, cultural and psycholinguistic barriers were identified, including issues pertaining to familiarity and semantic proportionality of stimuli. When the authors of the adaptation process agreed on potential solutions to these issues, they sought the consent of the authors of the original MoCA-H before implementing these in the adapted version. Once these modifications were made, the instrument was reviewed by a Portuguese language teacher.

### Stage 3 - Semantic analysis of stimuli

These procedures were performed remotely through Google Meet and involved 12 individuals, six male and six female, aged 28 to 56 years (M = 41.91, SD = 12.28), with nine to 16 years of education (M = 12.75, SD = 2.26), and no self-reported hearing or cognitive complaints.

Participants were selected by convenience through the personal connections of the researcher. This particular sample was not involved in any other stages of the study since its young adult participants were outside the target population for the MoCA-H. The aim of this procedure was to allow for a more critical assessment of the instrument by a younger population who was also more cognitively active and highly educated and could make additional recommendations on how best to adjust the instrument to the target population.

All participants in this sample were informed of the goals and procedures of this study upon being invited to participate. After agreeing to take part, individuals were sent an informed consent form and a Google Meet link through e-mail or WhatsApp messenger, providing them access to the non-expert assessment session.

The session was recorded and the instructions and application cards for the MoCA-H were shown to participants through screen sharing. Participants were encouraged to comment on the clarity of tasks and stimuli and asked how they would respond to each item in an assessment scenario, to verify that they understood what was asked and knew how to answer it. No cues or choice alternatives were provided to participants. The sample was also asked to read the task instructions and explain them in their own words. The duration of instrument application in this stage was 30 to 40 minutes. All information collected was entered into a table and descriptively analyzed with help from the authors of the original instrument.

### Stage 4 - Assessment by non-expert judges, part 1

This stage was also performed remotely through Google Meet and involved five non-expert judges, including three women and two men, aged 63 to 80 years (M=69, SD=6.63), with 12.5 to 16 years of education (M=15.2, SD=1.52), and no hearing or cognitive complaints. Participants were selected by convenience and inclusion criteria were evaluated by self-report.

Participants were contacted by the researcher through the WhatsApp messaging app and invited to take part in the study, inquired as to their availability to assess the application cards, and screened for inclusion criteria.

Raters also received an informed consent form and meeting link through WhatsApp. At the start of the meeting, the researcher read the informed consent form and confirmed the participants’ interest in entering the study before initiating the assessment process. The meeting had been previously scheduled and was fully recorded. The application cards were shown by the main researcher through screen sharing and after viewing each card, the judges were asked to rate the material as adequate or inadequate and indicate whether they were familiar with the concepts shown. The duration of administration was 20 to 30 minutes.

The non-expert judge ratings were entered into a spreadsheet and analyzed using Gwet’s AC1 coefficient as well as the content validity ratio (CVR) per item and cognitive domain.

### Stage 5 - Assessment by non-expert judges, part 2

Since the previous stage of the study identified a need for further modifications of the instrument, a new analysis was conducted to ensure the clarity of the proposed changes. This analysis was carried out by a non-expert 63-year-old male judge with 11 years of education and no self-reported hearing or cognitive complaints. This individual had not been involved in any other stage of the study, which allowed for a more accurate analysis since their first contact with the instrument occurred after the modifications had already taken place.

The rater was contacted via WhatsApp and invited to participate in the study. Once he received the informed consent form and agreed to participate, a Google Meet session was scheduled, performed, and recorded with the participant’s consent. The judge was then asked to rate each card as adequate or inadequate, as performed in the previous stage of this study. The test lasted approximately 20 minutes.

The rater evaluated all tasks, including those identified as inadequate by previous raters, and did not report any difficulties. At this point, the authors agreed that this stage was completed. The data were analyzed using descriptive methods.

### Stage 6 - Pilot study

This stage of the study occurred in person and was performed in the Speech Pathology Service of a public university in southern Brazil.

The study was advertised through social networks, family and professional contacts, waiting lists, and direct contact with patients in the hearing aid department of the aforementioned service.

The study included participants aged 60 years and older; with no sign of cognitive decline as assessed by the Mini Mental State Examination (MMSE)^(46)^; at least four years of education; a pure tone average (0.5kHz, 1kHz, 2kHz and 4kHz) within the normal range (<20dB) or bilateral hearing loss, symmetric or asymmetric, moderate (35-49 dB), moderately-severe (50-64 dB) or severe (65-79dB)^(47)^ with open-set speech comprehension, bilateral hearing aids for at least six months and at least six hours of daily hearing aid use (confirmed by self-report, patient records and/or data logging).

The MMSE was selected as a screening tool over the MoCA to avoid learning effects that would interfere with the results of this study since the standard version of the instrument is very similar to the MoCA-H. Since the MMSE involves oral instructions, all participants with hearing loss wore bilateral hearing aids during its administration, and the examiners used compensatory strategies (hyperarticulated speech, sitting face-to-face with the patient in a well-lit environment) whenever necessary.

The application of eligibility criteria led to the selection of 30 participants, 15 with normal hearing (PWNH) and 15 with bilateral moderate to severe hearing loss (pure tone average equal to or greater than 35dB - WHO^(47)^ (PWHL). Individuals with: mild, profound or untreated bilateral hearing loss; less than six daily hours of hearing aid use; less than six months total with hearing aids; MMSE scores indicative of cognitive decline, were excluded (n=23 individuals). Table 1 shows the sociodemographic characteristics of each group.

**Table 1:** Sociodemographic Characteristics of Each Group

Group	N	Sex	Age	Scholing	PTA RE	PTA LE
F/M	Med (SD)	Med (SD)	Med (SD)	Med (SD)
PWHL	15	7/8	72.13 (6.51)	9.06 (5.76)	60.25 (18.26)	51.58 (8.69)
PNHL	15	10/5	64.73 (2.89)	7.3 (3.85)	9.33 (5.15)	9.91 (5.62)

Caption: PWHL = Participants with hearing loss; PNHL = Participants with no hearing loss; F = Female; M = Male; Med = Media; SD = Standard Deviation; RE = Right ear; LE = Left ear; PTA = Pure Tone Average

This investigation was conducted across two cities in southern Brazil by a speech pathologist and a speech pathology undergraduate student, both of whom had been trained and certified in the administration of the instrument. Assessments took place over a single session conducted in a silent place with an average duration of two hours.

During the administration of the MoCA-H, participants read each of the 77 application cards out loud and followed the instructions for each stage of the test. The cards were printed in size A4, 300g layer paper with horizontal instructions, and were presented to participants one at a time. The following materials were used in the administration of the instrument: a ballpoint pen, clipboard, watch, black marker, paper, alcohol, and face shield.

For the first three tasks, individuals were given a pen and the test form so they could respond to all items with no interference from the examiner. All remaining tasks were answered orally using the instruction cards. The instrument allows for the assessment of eight cognitive domains: visuospatial/executive skills, naming, memory, attention, language, abstract reasoning, late recall, and orientation.

The visuospatial/executive domain includes three tasks that assess cognitive flexibility and inhibition (executive functions), planning, and constructive apraxia. Language is evaluated through the naming of three animal drawings, letter F fluency, and the construction of two sentences. Abstract reasoning is investigated using word categorization. Memory is assessed through the recall of a five-word list. Attention is assessed through forward and backward digit span, mental calculation, and vigilance tests. Spatial and temporal orientation are investigated by asking the patient to inform the date and location of the assessment session. The application ranged from 20 to 30 minutes depending on the difficulties presented by participants as a result of their education level.

Researchers were trained and certified in the application of the instruments. In addition to receiving training on the administration and interpretation of the standard English-language version of the instrument, researchers used the MoCA-H instruction manual to ensure the instrument was correctly administered.

Lastly, all participants who presented with auditory or cognitive alterations received appropriate orientations and referrals.

All data compiled in this stage of the study were descriptively analyzed.

## RESULTS

### Stage 1 - Translation and back translation of the MoCA-H

The two independent translations were compared by the authors of the adaptation process who identified no content differences between them. While some words differed between the two versions, all were synonymous and did not influence test comprehension (e.g., “Copy this drawing as accurately as you can” and “Copy this drawing in the most accurate way possible”). Once the translations were combined, back translation was performed and the resulting document was sent to the original authors who approved this version of the text.

### Stage 2 - Stimulus analysis and selection

The translated stimuli and instructions were analyzed and some questions were raised and answered by the authors. The questions and answers are shown in Chart 2 together with the tasks to which they correspond and their respective instruction cards.

**Chart 2:** Questions regarding tasks in the MoCA-HI answered by authors

Task	Card (instruction)/task	Question	Answer
Visuospatial/executive functions	Clock task	The phrase “10 past 11” could not be translated literally since this expression does not exist in Brazilian Portuguese. The closest to this would be “10 to 11.” Can we use that instead? We also believe that different cognitive processes would be required to understand this expression as opposed to “10h 50 min.”	You should use “eleven ten.” This is what we used in other translations. No It would be as if we were “tricking” the subject by wording the instructions as “10 past 11.”
Memory	The examiner shows card (8) which contains the following text: “This is a memory test. I will show you a list of words that you will have to remember now as well as later. Read it carefully. When I am finished, tell me how many words you remember. The order in which you say them does not matter.”	- Does the phrase “When I finish” refer to the joint reading by the patient and examiner or to the end of the instructions?	“It refers to the moment at which the researcher finishes showing the words.”
Verbal Fluency	The examiner shows card (28) which contains the following text: “Now I want you to say as many words as you can starting with the letter F.” I will ask you to stop after one minute. Proper nouns, numbers, and different forms of a single verb are not allowed. Are you ready?” [Pause] [Set timer for 60 seconds]. When the timer gets to 0, the examiner shows card (29): “Stop.” If the subject elicits two consecutive words that begin with another letter of the alphabet, the examiner shows card (28) once again and points to the target letter if the instructions have not yet been repeated.	How many times can the instructions be repeated?	All instructions can only be repeated once. However, if the instructions were repeated and the individual elicits words that do not start with the letter named in the instructions (F), the examiner can show card (28) again.
Abstract Reasoning	“Now, a train and a bicycle” (card 35). Once the patient answers, the examiner presents the second stimulus, showing card (36). “Now, a ruler and a watch.” Cue card (33): a single cue card is available for the entire abstract reasoning section and can be shown if none were used during the example.	Can we show card 33 (cue card) at any point during the abstract reasoning tasks?	Yes, the cue card containing the sentence “Tell me another category to which it belongs” can be used only once in this section.

Additional stimulus modifications were also suggested, such as replacing “daisy” with “rose,” since the latter is more familiar to the Brazilian population and cited in nursery rhymes much like “daisy” in English. The word “red” (“*vermelho*”) was changed to “blue” (“*azul*”) due to the length of the word in Portuguese relative to the word “red.” Lastly, the multiple-choice option “daffodil” was replaced by “violet” which is also more common in Brazil.

### Stage 3 - Semantic analysis of stimuli

Several tasks in the first version of the instrument required adjustments in subsequent stages. Chart 3 shows the difficulties identified in the instrument and any changes made to address them.

**Chart 3:** Cards identified as inadequate by the semantic analysis

Inadequate card	Instruction	Difficulty	Modification
2	Please draw a line from a number to a letter in ascending order. Start with (1) and draw a line from 1 to A, then to 2, and so on. End at (E).	Understanding the switch between letters and numbers.	No
9^IV^	Rose	The word “rose” could refer to a flower or the color pink (the word “*rosa*” has both meanings in Portuguese)	Yes, the plural form “roses” was used to prevent misunderstandings
36	Now, a ruler and a watch	Ruler-watch not considered to belong to the same category	No
40^V^	Now tell me the name of this place and the city it is in.	Understanding that the card asks the respondent to identify their location at the time of the assessment.	Yes

A specific change was suggested in card 36 (changing ruler-watch to ruler-timer) by a participant in this stage. However, the change was not authorized by the authors of the original instrument out of concern that it would alter the task’s difficulty level. Therefore, the original words were retained.

### Stage 4 - Assessment by non-expert judges, part 1

Chart 4 presents the cards identified as inadequate by non-expert judges and modifications made in response to their observations.

**Chart 4:** Cards identified as inadequate by non-expert judges, part 1

Inadequate Card	Instruction	Difficulty	Modification	Details of the modification
2	Please draw a line from a number to a letter in ascending order. Start with (1) and draw a line from 1 to A, then to 2, and so on. End at (E).	Understanding the switch between letters and numbers.	Yes	In the Portuguese version, the sentence was reorganized and the term “going” was removed and the term “alternating” was inserted. “From A to 2” was added.
8	This is a memory test. I will show you a list of words that you will have to remember now as well as later. Read carefully. When I finish showing the list, tell me as many words as you can remember.	In the task instructions	Yes	The first sentence of the third paragraph has been rearranged.
30	Now I will show you two words. I would like you to tell me what category they belong to.	Understanding what a category is	No	No change
31	An orange and a banana	Understanding what a category is	No	No change
36	Now, a ruler and a watch	Ruler-watch not considered to belong to the same category	No	No change
40	Tell me what day it is today.	Does not find it adequate, suggests date (eg. DD/MM/YY).	Yes	The term “day” was substituted by “date”
40^iv^	Now tell me the date	Inadequate, suggests date (eg. DD/MM/YY).	No	No change
40^V^	Now tell me the name of this place and the city it is in.	Understanding that the card asks	Yes	We changed the part “this place” to “place where you
Inadequate Card	Instruction	Difficulty the respondent to identify their location at the time of the assessment.	Modification	Details of the modification are” and the part “which city is it in” to “city where you are”

The agreement between the five non-specialist judges regarding each task of the MoCA-H was then analyzed (Table 2).

**Table 2:** Agreement Between Non-Specialist Judges Regarding MOCA-HI Items

Cards	Cognitive Domains	CVR	AC1/Cognitive Domain/	AC1/Total
CARD 1	Visuospatial/executive function	1	0.817	0.939
CARD 2		0.2		
CARD 3		1	[CI = 0.121-1]	
CARD 4		1		
CARD 5	Naming	1	1	
CARD 6		1		
			[CI = 1-1]	
CARD 7		1		
CARD 8	Memory	0.6	0.973	
CARD 9i		1		
CARD 9ii		1		
CARD 9iii		1		
CARD 9iv		1		
CARD 9v		1		
CARD 10		1		
CARD 11		1	[CI = 0.912-1]	
CARD 12i		1		
CARD 12ii		1		[CI = 0.895-0.984]
CARD 12iii		1		
CARD 12iv		1		
CARD 12v		1		
CARD 13		1		
CARD 14		1		
CARD 15	Attention	1	1	
CARD 16i		1		
CARD 16ii		1		
CARD 16iii		1		
CARD 16iv		1		
CARD 16v		1		
CARD 17		1	[CI = 1-1]	
CARD 18i		1		
CARD 18ii		1		
CARD 18iii		1		
CARD 19		1		
CARD 20		1		
CARD 21	Attention	1	1	0.939
CARD 22		1		
CARD 22		1	[CI = 1-1]	
CARD 23		1		
CARD 24	Language	1	1	
CARD 25		1		
CARD 26		1		
CARD 27		1	[CI = 1-1]	
CARD 28		1		
CARD 29		1		
CARD 30	Abstract Reasoning	0.6	0.749	
CARD 31		0.6		
CARD 32		1		
CARD 33		1	[CI = 0.380-1]	
CARD 34		1		
CARD 35		1		
CARD 36		0.2		[CI = 0.895-0.984]
CARD 37	Late Recall	1	1	
CARD 38		0.2		
CARD 39i		1		
CARD 39ii		1		
CARD 39iii		1		
CARD 39iv		1		
CARD 39v		1	[CI = 1-1]	
CARD 39vi		1		
CARD 39vii		1		
CARD 39viii		1		
CARD 39ix		1		
CARD 39x		1		
CARD 40i	Orientation	0.6	0.617	
CARD 40ii		1		
CARD 40iii		0.6	[CI = 0.016-1]	
CARD 40iv		0.2		

Caption: CVR = Content Validity Ratio; AC1 = *Gwets* AC1; CI = Confidence interval

At this point, the researchers also examined the translation of card 28 which describes the types of words that are not permitted in the letter “F” fluency task, such as numbers. This led to the removal of the word “numbers” from the instruction since, unlike English (e.g., “five”), Portuguese has no numbers starting with “F”.

### Stage 5 - Assessment by non-expert judges, part 2

In order to ensure that the modifications suggested in the analysis of the previous stage (Stage 4) have brought clarity to the instrument, the authors have decided to carry out the application of the instrument on a new subject. All cards were rated as adequate by the judge involved in this stage, and no other difficulties or observations were identified. The instrument was therefore printed out for use in the subsequent stages of the study.

### Stage 6 - Pilot study

In order to standardize the administration process, all authors involved in the adaptation agreed on the development of a complementary instruction card to be used at the start of instrument administration. It contains the following information: “Attention! In a few minutes, some blank cards will be shown. These indicate that the examiner is awaiting your response or reaction to the request in the previous card.”

At this point, the cue cards for the target words in the late recall task were laminated and, depending on the responses of each participant, multiple choice alternatives for these items would be written down by one of the researchers. The duration of application at this point ranged from 20 to 30 minutes.

The researchers also found that when participants read the instruction cards aloud, they were more worried about their reading fluency than the content of the cards. This led to execution errors in some tasks with participants responding immediately, without reading the full instructions on the card.

Another observation made by the researchers was that after reading some instruction cards, the subjects tended to respond to subsequent cards based on this instruction rather than reading the new cards as well. When the researchers noticed this behavior, they would point to the card with their index finger and emphasize the orientation of reading the card before providing an answer. This was especially common in cards five, six, and seven (naming tasks), 19 and 21 (attention), and 25 (language).

Participants in this stage had more difficulty in tasks involving visuospatial skills (trails: 22 errors, cube: 17 errors), language (1st sentence: 12 errors, 2nd sentence: 15 errors), attention (vigilance: 12 errors), and abstract reasoning (ruler-watch: 15 errors).

## DISCUSSION

In recent years, a growing number of international instruments have been adapted into BP^(48-52)^. One of the advantages of adaptation over the creation of a brand-new instrument is the speed of the process since existing instruments already have well-defined constructs and assessment methods (items). Yet each process has its own set of strict guidelines, involving different populations and methodological procedures^(53,54)^.

The English language MoCA-H^(43)^ was adapted from the standard MoCA, which was also originally published in English^(16)^. The adapted version was developed when researchers noticed the effect that speech comprehension difficulties faced by individuals with hearing loss had on the administration of the MoCA. Since this is an orally administered instrument, hearing impairments could interfere with test results. To address this issue, it was crucial to modify the mode of application. In the MoCA-H all instructions are presented on cards that must be read aloud by the respondent to prevent the interference of hearing impairments on test answers^(43)^. This instrument is available in English, Dutch, German, and Italian^(55)^.

Given the impact of hearing loss on cognitive skills^(4,5,56,57)^, the present study sought to offer professionals and researchers an instrument that would allow for the cognitive assessment of BP speaking older adults with hearing loss. The MoCA-H could make a significant contribution to this area of study, and as such, we followed all necessary steps to adapt the instrument from English to BP^(58,59)^.

One of the first stages of the present study involved the development of independent translations of the instrument, always ensuring that its original features were preserved and that more than one translator was involved to establish cultural and conceptual consistency and preserve the meaning of all terms in the original version^(60)^. A back-translation procedure was also carried out, and its result was submitted for assessment and approval by the authors of the original instrument. This is an essential part of the adaptation process and should consider cultural, linguistic, idiomatic, and contextual characteristics to help ensure conceptual equivalence between versions^(61)^.

Semantic analysis is also essential for instrument adaptation, since some of the translated terms and phrases may not be common in the target population (stage 3), as was the case of “daisy” in the present study, which was replaced by “rose.” However, this was a rare exception, since the vast majority of words in the original instrument were known to Brazilian respondents.

Still in the semantic analysis stage, given the lack of clarity in the request for location identification (Charts 3 and 4) in card 40^V^, the sentence structure had to be adjusted to ensure the item was clear and suited to its adapted, as expected when all steps involved in an adaptation process are rigorously followed^(61)^. Participants in this stage of the study also had difficulty with visuospatial/executive (alternate trails test) and abstract reasoning tasks (categorization of “ruler” and “watch”). In response to the latter, one participant suggested that the word “watch” be replaced by “timer,” but according to the authors of the original instrument, this change would affect the difficulty of the task and therefore could not be implemented. Another study also observed a similar issue and changed the term “watch” to “scale^”(62)^. This possibility was not considered in the Portuguese version of the instrument or suggested by the authors of the original MoCA-H.

Psycholinguistic criteria were also assessed in the present study (Stage 4) to identify the need for changes related to linguistic context since cross-cultural adaptations of assessment instruments often require such adjustments. To ensure the validity of an instrument, for instance, its words and verbal stimuli must be familiar to respondents^(62,63)^. This was the reason why, as previously mentioned, the word “daisy” was changed to “rose” in this study. However, this was in itself ambiguous since the word “rosa” in Portuguese may mean both the flower and the color pink. To avoid confusion, the word was then changed to “roses” (Chart 3). A similar change was made by the authors of the Philippine version of the standard MoCA, where the word *“daisy”* was altered to “roses” given the lack of familiarity of the target population with this term. The abstract reasoning task (categorization of the words “ruler” and “watch”) was also found to be difficult by responders in this study^(62)^.

The pilot study revealed that the difficulties in measures of executive functions (alternate trails) and abstract reasoning (ruler and watch) remained since both were complex for subjects across both participant groups since the semantic analysis stage. In other tasks, however, no difficulties were observed after the adaptations. The difficulties in the visuospatial/executive tasks in the present study may be attributable to the cognitive effects of aging since the first brain regions affected by age-related neurodegeneration are the frontal lobes, which are responsible for the executive functions^(64,65)^.

The pilot study is crucial to determine the length of application and identify the need for any additional adjustments in the instrument. Studies show that this stage of real-world testing can reveal issues that were not identified in earlier stages of the adaptation process^(66,67)^. The pilot study also provides an opportunity to assess respondents’ comprehension of stimuli and instructions^(67)^ and identify the need to add, modify or complement the changes made to the instrument, all of which constitute a natural part of the adaptation process.

Therefore, even after modifications were made to improve task comprehension, some items may still be perceived as difficult for participants. It is important to note that the changes made to the instrument during the cross-cultural adaptation process cannot compromise item comprehensibility^(68)^.

We encourage future studies to examine the performance of individuals with hearing loss and no use of HADs on the MoCA-H to verify whether their scores differ from those of individuals with no hearing loss or cognitive impairment. We also underscore the need for psychometric studies to collect evidence of construct validity, reliability, sensitivity, and specificity for this instrument.

## CONCLUSIONS

The present study was successful in adapting the MoCA-H into BP yielding an instrument with satisfactory content validity. Additional studies should be performed to collect further evidence of the instrument’s validity. The instrument version is now available at the link in MoCA Cognition^(55)^.
